# The structured health intervention for truckers (SHIFT) cluster randomised controlled trial: a mixed methods process evaluation

**DOI:** 10.1186/s12966-022-01316-x

**Published:** 2022-07-07

**Authors:** Amber J. Guest, Nicola J. Paine, Yu-Ling Chen, Anna Chalkley, Fehmidah Munir, Charlotte L. Edwardson, Laura J. Gray, Vicki Johnson, Katharina Ruettger, Mohsen Sayyah, Aron Sherry, Jacqui Troughton, Veronica Varela-Mato, Thomas Yates, James King, Stacy A. Clemes

**Affiliations:** 1 School of Sport, Exercise and Health Sciences, Loughborough University, Loughborough, Leicestershire, LE11 3TU UK; 2 NIHR Leicester Biomedical Research Centre, Leicester, UK; 3 Centre for Physically Active Learning, Western Norway University of Applied Sciences, Bergen, Norway; 4 Wolfson Centre for Applied Research, Faculty of Health Studies, University of Bradford, Bradford, UK; 5 Diabetes Research Centre, University of Leicester, Leicester, UK; 6 Department of Health Sciences, University of Leicester, Leicester, UK; 7 Leicester Diabetes Centre, University Hospitals of Leicester NHS Trust, Leicester, UK

**Keywords:** Workplace, Physical activity, Sedentary behaviour, Dietary behaviours, Occupational health, Truck drivers, Process evaluation, Behaviour change, Healthy lifestyle

## Abstract

**Background:**

This paper presents the mixed methods process evaluation of the randomised controlled trial (RCT) of the Structured Health Intervention For Truckers (SHIFT), a multi-component intervention targeting physical activity and positive lifestyle behaviours in a cohort of 382 truck drivers in the UK. The SHIFT RCT found a significant difference in daily steps between intervention and control groups at 6-months in favour of the intervention participants.

**Methods:**

SHIFT was evaluated within a cluster-RCT and involved 25 transport sites (12 intervention and 13 control sites). Intervention components included an education session, Fitbit, text messages, and cab workout equipment. Participants completed questionnaires at baseline and 6-months follow-up. Semi-structured focus groups/interviews were conducted with drivers (*n* = 19) and managers (*n* = 18) from each site, after completion of the final follow-up health assessment (16-18 months post-randomisation). Questionnaires and interviews collected information on fidelity, dose, context, implementation, barriers, sustainability, and contamination.

**Results:**

Questionnaire and interview data from intervention participants indicated favourable attitudes towards SHIFT, specifically towards the Fitbit with a high proportion of drivers reporting regularly using it (89.1%). 79.2% of intervention participants attended the education session, which was deemed useful for facilitating improvements in knowledge and behaviour change, dietary changes were predominantly recalled. Despite not being part of the intervention, participants reported that feedback from the health assessments motivated them to change aspects of their lifestyle (intervention = 91.1%, control = 67.5%). The cab workout equipment was used less and spoken unfavourably of in the interviews. The main barriers to a healthy lifestyle at work were reported as long hours and irregular shift patterns. The most suggested improvement for the intervention was more frequent contact with drivers. Managers were positive about the objectives of SHIFT, however almost all mentioned the challenges related to implementation, specifically in smaller sites.

**Conclusions:**

Overall, SHIFT was predominantly implemented as intended, with minimal discrepancies seen between the delivery and protocol. Having said this, transport sites each have distinct characteristics, which may require adaptations to individual settings to encourage participation. Managers and drivers reported enthusiasm and necessity for SHIFT to be included in future Certificate of Professional Competence training.

**Trial registration:**

ISRCTN10483894 (date registered: 01/03/2017).

## Background

Truck driving encompasses a variety of inherent characteristics which create an unhealthy working environment, such as chronic time pressure, poor truck-stop facilities, sleep deprivation, high demand, low control, enforced sedentary behaviour, and long, irregular working hours [1]. As a result, truck drivers often display worse health profiles than the general population, including a higher prevalence of obesity, hypertension and mental ill-health, alongside low physical activity, and poor dietary habits [2]. The health of truck drivers is of public concern given that poor health leads to increased risk of collisions [3–7]. In addition, truck drivers are an ageing population [8], and the UK Logistics sector has been described as experiencing a ‘demographic time-bomb’ [9]. These factors are contributing to an increasing shortage of truck drivers, approximately 100,000 in the UK alone, putting extreme pressures on supply chains and economies [8]. To date, limited health behaviour interventions within truck drivers have been conducted, and of those which have, the evidence is limited by small sample sizes, pre-post study designs and no follow-up periods [10]. It is timely therefore to explore how large interventions could be delivered which aim to address some of the health barriers and unintended health consequences inherent within this profession.

The Structured Health Intervention for Truckers (SHIFT) is a multi-component, theory driven, health behaviour intervention designed to promote physical activity and positive lifestyle changes in truck drivers. Full details of the intervention and trial protocol have been reported elsewhere [11, 12]. The intervention consisted of an interactive, group-based 6-hour education session, grounded within the Social Cognitive Theory (SCT) for behaviour change [13, 14], delivered by two trained facilitators in the drivers’ workplace. Participants were provided with a Fitbit Charge 2, designed to provide them with the real-time feedback on their activity levels. Individually tailored ‘step count challenges’ were facilitated at 6-weekly intervals throughout the 6-month intervention, via a text messaging service. Participants were also provided with equipment to do a ‘cab workout’ whilst waiting at delivery stops. Throughout the intervention, participants received health coach support from members of the research team (via text messages), and from trained worksite champions (fellow drivers who were driver trainers, trained by the research team to co-facilitate the education sessions, and act as worksite champions).

The intervention was implemented across 25 transport sites of a major Logistics company and evaluated using a cluster randomised controlled trial (RCT). Baseline health measurements were conducted prior to randomisation and included a 2-hour health assessment, involving a range of anthropometric, cardiometabolic health measures, and a number of self-reported measures of wellbeing and work-related psychosocial variables. All participants received detailed feedback on their health measures during this assessment. Following the health assessment, participants were provided with two devices (an activPAL and a GENEActiv) to provide device-based measures of physical activity (step counts were the primary outcome in the RCT), sitting time and sleep. Health assessments were repeated at 6-months follow-up, and a final set of measures took place at 16-18 months post-randomisation. These final measures were delayed due to the COVID-19 pandemic and consisted of the self-report measure and use of the activPAL only. At 6-month follow-up, the intervention group did 1008 steps/day more than the control arm. This was primarily due to the control group reducing their mean step count at 6-months compared to baseline, and the intervention group remaining stable. The study found no differences in BMI, dietary intake, or blood biomarkers between groups [12].

The purpose of this process evaluation was to understand the key elements comprising implementation fidelity, dosage, attrition rates, contamination, barriers, context, and intervention sustainability. Further aims were to describe and understand the contextual factors associated with both the intervention and the evaluation of it within a cluster RCT, and to provide recommendations for refinement of the programme for future sustainability.

## Methods

**Fig. 1:**
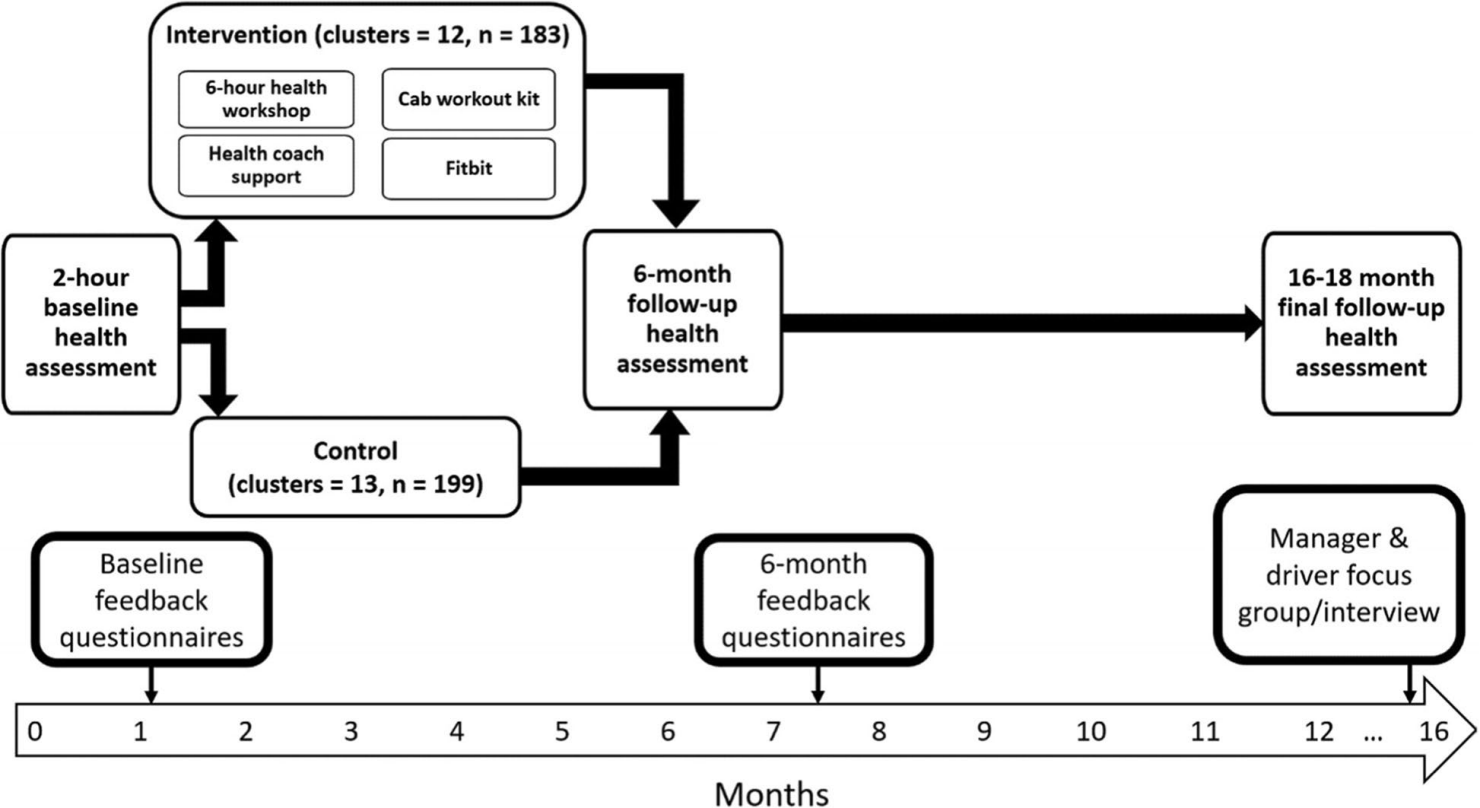
Schematic of the SHIFT programme and integrated process evaluation

Three-hundred and eighty-two truck drivers were enrolled into the study at baseline, recruited across 25 transport sites (i.e., clusters). Sites were randomised into the two trial arms following the completion of baseline measures. The participants at intervention sites (sites = 12, *n =* 183) received the SHIFT programme, whilst the participants at control sites (sites = 13, *n =* 199) were advised to continue as usual. A schematic of the combined SHIFT project and process evaluation can be seen in Fig. 1.

Recruitment began in August 2017 with an internal pilot phase which involved the first six sites. Baseline data collection occurred between January and August 2018, and follow-up data collection occurred between September 2018 and November 2019. Recruitment for the main trial phase began in January 2019, baseline data collection occurred between February and July 2019, and follow-up data collection occurred between September 2019 and December 2020. Table 1 shows a summary of sample characteristics at baseline.

**Table 1:** Baseline sample characteristics

Measurement	N Total = 382	Mean (standard deviation (SD)) Median (interquartile range (IQR)) * N (%)
Age (years)	380	48.4 (9.4)
Gender (n, % men)	382	378 (99.0%)
Ethnicity (n, % White British)	382	306 (80.1%)
Duration working as truck driver (years)	382	16.0 (9.0, 25.2) *
Average working hours (hours/week)	381	48.0 (5.0) *
BMI (kg/m^2^)	380	29.8 (26.9, 33.2) *
Systolic blood pressure (mmHg)	380	130 (112, 139) *
Diastolic blood pressure (mmHg)	380	82 (76, 88) *
Heart rate (bpm)	378	68 (10)
Sleep duration (minutes/day)	346	371 (337, 406) *
Sitting (minutes/day)	341	665 (95)
Steps (steps/day)	329	8592 (6964, 10,704) *
MVPA (minutes/day)	341	10 (14) *
Cluster size (number of participants per depot)	382	14 (13, 17) *

### Process evaluation

The Medical Research Council (MRC) framework was used to guide this process evaluation [15] through assessing three key components: implementation, mechanisms of impact, and context. Implementation was measured through capturing fidelity, dose, and adaptations made to the RCT. Mechanisms of impact was measured by firstly understanding the participant responsiveness, and secondly testing the logic model created as part of the SHIFT protocol. Context was measured throughout the RCT, to determine contextual factors that affect implementation, intervention mechanisms, and outcomes. Mixed methods were used to deepen analytic understanding of a specific issue and in turn triangulate results [16]. Fidelity and dose were measured both quantitatively and qualitatively. Qualitative research techniques were used to identify how context affects implementation, barriers, mechanisms of impact, and future sustainability. Implementation outcomes were examined using a feedback questionnaire in combination with in-depth interviews and focus groups, with drivers and the main liaison managers from control and intervention sites. Table 2 summarises each of the evaluation outcomes examined, how they were defined and how they were measured.

**Table 2:** Summary of the process evaluation measures used

Implementation outcome	Definition	Data source	Time point
Context of the intervention	Contextual factors that impacted the implementation, intervention mechanisms, and outcomes	Initial discussions with site managers prior to intervention implementation	Pre-study
		Focus groups/interviews with drivers and managers	Final follow-up
		Researcher field notes	Continuous
Fidelity	The extent to which the intervention was delivered as planned	Internal monitoring of project attendance records	Post-study
		Focus groups/interviews with drivers and managers	Final follow-up
Dose delivered/ received	Dose delivered: the amount of the intervention that was provided by the SHIFT team Dose received: How frequently participants engaged with intervention components	Follow-up questionnaires	6-months
		Text Magic statistical reporting software (Cambridge, UK)	Continuous
		Internal monitoring of project attendance records	Post-study
Adaptations	Changes made to improve the delivery of the intervention	Focus groups/interviews with the drivers and managers	Final follow-up
		Fortnightly reflective research team meetings	Continuous
Sustainability	Maintenance of health behaviours following the 6-month intervention period The extent to which drivers and managers envisaged SHIFT becoming sustainable in the future	Focus groups/interviews with the drivers and managers	Final follow-up
Mechanisms of impact	Strategies put in place by intervention participants to facilitate behaviour change	Focus groups/interviews with the drivers and managers	Final follow-up
		Follow-up questionnaires	6-months
Contamination	Interactions between intervention and control sites	Focus groups/interviews with the drivers and managers	Final follow-up

### Data collection

#### Project records

Records were gathered from all participants to inform dosage and attrition rates, including: participation uptake per site (number of participants who completed the baseline health assessment per site); dropout rate per site (number of participants who failed to complete each follow-up); attendance at the education session; engagement with text messaging service (number of messages sent and number of responses); and compliance with activity monitors (≥1 valid day).

#### Questionnaires

All participants were asked to complete questionnaires 1 month after the baseline health assessment and 1 month after the six-month follow-up health assessment, using a combination of multiple choice, open-ended, and Likert scale questions. At baseline, the questionnaires sought feedback about the baseline health assessment (although not part of the intervention, previous research has shown that being measured as part of the evaluation can have an impact on participants’ lifestyle [17]. The intervention follow-up questionnaire sought feedback about the quality, dose, and usefulness of each component of SHIFT (i.e., the education sessions, cab workout equipment, Fitbit, step challenges, text message feedback), and any other lifestyle or work changes that may have impacted the results. Control participants were asked about both baseline and follow-up health assessments and any other lifestyle or work changes that may have impacted the results. Content analysis was used to quantify open-ended questions by listing key responses into themes and calculating frequencies in each theme [18].

#### Focus groups and interviews

Managers from pilot sites were initially asked to recruit and schedule in three to six drivers for an on-site focus group during the drivers’ working hours, based on availability during the drivers’ work schedule. A semi-structured focus group schedule was developed and trialled with drivers from pilot sites (*n =* 6). The intervention participants were asked questions regarding each SHIFT component, barriers to achieving health goals, perceived benefits and experiences, and recommendations for improvements to SHIFT. The control group were asked questions about contamination between intervention and control sites, and any lifestyle changes that occurred during the study. The main liaison manager from each site was also interviewed on the same day. These interview schedules were then analysed and revised as needed before commencing data collection for the main trial sites (*n =* 19).

Due to the outbreak of SARS-CoV-2 and a national lockdown (March 2020 – July 2021), in-person focus groups were no longer feasible, and therefore telephone interviews were completed for main trial sites. A purposive sampling approach was used to yield information rich cases relating to the implementation of SHIFT in practice, this included the main liaison manager and one driver from each depot. As these interviews occurred during non-work hours, all drivers were texted to ask if they would be interested in participating, with the offer of a £5 high street voucher and a chance to win a Fitbit. The main liaison manager from each site was recruited via email and interviewed via telephone. Table 3 shows the data collected through each method.

**Table 3:** Data collected from drivers and managers

	Intervention participants				Control participants			
	Baseline questionnaire (*n =* 113)	6-month questionnaire (*n =* 70)	Interview/ focus groups with drivers (*n =* 16)	Interview with managers (*n =* 9)	Baseline questionnaire (*n =* 160)	6-month questionnaire (*n =* 100)	Interview/ focus groups with drivers (*n =* 12)	Interview with managers (*n =* 9)
Baseline and Follow-up Health Assessments								
Did it encourage initial participation?	✓	✓			✓	✓		
How did it compare to expectations?	✓		✓		✓		✓	
Did awareness of health change?	✓		✓		✓		✓	
How understandable were the results?	✓		✓		✓		✓	
Most/least preferred measurements?	✓		✓		✓		✓	
Did motivation towards improving lifestyle change?		✓	✓			✓	✓	
Education Session								
How understandable was it?		✓	✓					
How engaging was it?		✓	✓					
How informative was the booklet?		✓						
Did awareness of health change?		✓	✓					
Did it impact motivation for lifestyle change?		✓	✓					
Were action plans created?		✓						
Were there any key messages?		✓						
Cab Workout								
Regularity of use?		✓	✓					
What was the most/least commonly used equipment?		✓	✓					
Where was the most/least common location for use?		✓	✓					
Potential barriers of cab workout?		✓	✓					
Fitbit								
How understandable was it?		✓	✓					
Did awareness of health change?		✓	✓					
Did it impact motivation for lifestyle change?		✓	✓					
Step Challenges								
Did awareness of daily steps change?		✓	✓					
Did it impact motivation for change in step count?		✓	✓					
Text Messages								
How was the frequency of text messages?		✓	✓					
How relevant was the content?		✓	✓					
Did it impact motivation for lifestyle change?		✓	✓					
How supported did participants feel?		✓	✓					
How efficient were the text messages?		✓	✓					
General								
How was the workplace managerial support?			✓	✓			✓	✓
Has awareness of health changed since commencing the study? If so, how?			✓				✓	
Has lifestyle changed since commencing the study? If so, how?			✓				✓	
If so, have changes fluctuated throughout the study?			✓					
Are there any barriers to a healthy lifestyle?			✓					
Most/least important component of the intervention?			✓					
Future direction of SHIFT into a certificate of professional competence (CPC) module?			✓	✓				
Were there any external impacts on lifestyle (such as moving house)?		✓	✓			✓		✓
Were there any external fitness/diet tracking techniques used?		✓	✓			✓		
How much did the pandemic affect study participation?			✓	✓			✓	✓
Thoughts of being in the control group?							✓	✓
Was there any contamination between control and intervention?			✓	✓			✓	✓
What was the role of the manager?				✓				✓
Did the managers have outcomes they hoped to achieve?				✓				✓
To what extend were there operational difficulties that impacted the study?				✓				✓
Can you see SHIFT becoming sustainable in the future as a CPC module?		✓		✓				
Did you hear of any reasons for dropout?				✓				✓

### Data analysis

All focus groups and interviews were conducted by one researcher (AG) and overseen by a second researcher for the first ten interviews (YLC). They were recorded on a digital audio device before being transcribed verbatim into Microsoft Word (by AG) and inputted into NVIVO 12 (QSR International) software for analysis. Transcripts were then re-read, coded, and themes developed using the deductive method of thematic analysis (AG), where themes were already preconceived based upon the interview schedule and existing knowledge. This was followed by an inductive method where themes were identified entirely based on the data (AG) [19]. Each stage of the analysis was critically analysed from an informed external perspective (AC). Quantitative data including dose, attrition, and questionnaire data such as multiple choice and Likert scale answers were analysed in IBM SPSS v27 and reported as means and percentages. The results are summarised according to the key implementation outcomes listed in Table 2.

## Results

### Participants

All participants were given feedback questionnaires 1 month after the baseline health assessment (*n =* 272/382, 71.2% response rate) and 1 month after the six-month follow-up health assessment (*n =* 167/289, 57.8% response rate). Six focus group discussions with participants (*n =* 13, 5/6 sites) and eight interviews with managers (5/6 sites) were undertaken to capture their views and experiences of implementation during the internal pilot. Telephone interviews were conducted with drivers (14/17 sites) and with managers (13/17 sites) from main trial sites after completion of the main trial. There were no substantial differences in data between pilot and main trial sites in both the questionnaire, the interviews, and focus groups, therefore the results will show the combined findings from both pilot and main trial sites.

### Dose delivered and received

It was intended that intervention participants would receive 12-hours of in person contact over 12-months (comprising three 2-hour health assessments and one 6-hour education session). Of the 183 intervention participants, 145 (79.2%) took part in the education session. Barriers to attendance were due to logistical challenges of co-ordinating drivers together owing to operational requirements.

Control participants were to receive 6-hours of face-to-face contact consisting of three 2-hour health assessments. Due to the SARS-CoV-2 pandemic, a national lockdown prevented all main trial participants from receiving an in-person final follow-up health assessment at 12-months follow-up, which reduced in-person contact by 2-hours. Instead, the 12-month follow-up occurred at 16-18 months post-randomisation and utilised remote data collection, with participants self-reporting weight, wearing an activPAL for 8 days, and completing the same questionnaires as baseline.

#### Text messages

The average number of text messages sent to each driver was 20.2 throughout the duration of the study, with a 97.5% delivery rate. The average number of text messages received from each driver was 3.8 (an 18.8% response rate). Though engagement with the text messaging was poor, it appeared the overall use of text messages were relatively positive.

*“I haven’t really text backwards and forwards, but when you sent me the little challenges [text messages] like I thought good, yeah, I enjoyed that.”* [Intervention Driver – P06].

#### Cab workout equipment

All intervention participants who attended the education session were given cab workout equipment (resistance bands and balls, and a grip strength dynamometer), as per trial protocol. Fifty-nine percent of intervention participants said they had used their cab workout equipment in the last 6 months. Few planned to use it in the future. For those that did use it, the most used piece of equipment was the hand gripper, followed by the resistance bands. There was minimal reported use of the fitness ball. Of those who used the cab workout equipment, only 16.3% either agreed or strongly agreed it increased their physical activity levels.

*“Yeah, the hand gripper, I use that, the stretchy bands, I used to use them more, because I could tie it to the bottom of the handrail in the cab and I could exercise my arms, both arms … I’ve still got them, and I still use them … They’re alright, they do what they’re meant to do.”* [Intervention Driver – M14].

Reasons for low adherence levels were explained in more depth throughout the interviews. Some suggested the impracticality of the workout equipment in their cabs was a barrier to its use. Participants also indicated they prioritised sleeping and eating on their breaks over being physically active.

*“I don’t think it’s practical. You have 50 minutes break and by the time you’ve been to the toilet, you’ve had your dinner, you need a nap, if you’ve done three and a half, four hours of driving, you need shut eye for 10, 15 minutes. There’s just not enough time … To do those things.”* [Intervention Driver – M17].

Participants also suggested that there may be a sense of embarrassment for some drivers, particularly the older ones, when using the equipment.

*“I think with a lot of the old boys, like the drivers, maybe they wouldn’t want to do something like that because they’d feel stupid doing it to be honest … they’d have just been like ‘oh I’m not doing that’”* [Intervention Driver – P03].

#### Fitbit

All intervention participants who attended the education session were given a Fitbit and instructions on how to use it, as per trial protocol. The majority (89.1%) of intervention participants reported they still used their Fitbit at the 6-month follow-up, 7.3% had previously used it but no longer use it, whilst only 3.6% had never used it and did not intend to in the future. Those that did not use the Fitbit mainly said it was due to causing skin irritation, already having an activity monitor, or not knowing how to use the device.

*“I have no idea how to use the watch. I have no idea how to …*. *I mean, I’m hoping that this Fitbit thing is going to show me just how bone idle I am!”* [Intervention Driver – P03].

### Fidelity and adaptation

All health assessment measures for baseline and 6-month follow-up were adhered to throughout the study in line with the study protocol [11]. All health assessors received 4-hours of training on the standard operating procedures followed by two shadow shifts as per protocol. All baseline health assessments were conducted at the beginning of the drivers’ shift, as per protocol. However, this was logistically challenging for both the researchers and transport managers to facilitate. As a result, post-intervention measures were conducted at a time of day which was more feasible, and occasionally meant they took place at the end of the drivers’ shifts. Drivers were still asked and reminded to fast for 4-hours prior to the assessment, however this adaptation to the programme may have affected body weight and blood pressure measurements. Additionally, as previously mentioned, a national lockdown prevented all main trial participants from receiving an in-person final follow-up health assessment. These unforeseen events meant it was agreed the primary outcome measures were changed from 12-months to 6-months follow-up.

No discrepancies were seen between the protocol and the content delivery of the education session. All education sessions lasted 6-hours and were delivered by two trained educators, consisting of either two researchers, or one researcher and one driver trainer, all of whom were trained by the Leicester Diabetes Centre. The initial target was to include six participants per education session, however this was logistically challenging for the transport managers to take six drivers off the road at any one time, particularly for the smaller sites, and as a result, most education sessions consisted of fewer drivers per workshop (mean = 3.4, range 1-7). There were some logistical issues with organising the education sessions in some sites, with the average duration between baseline health assessment and education session being 4 months (range 2-7 months), which differed from the planned timeline of a one-month interval.

### Participant responsiveness

#### Education session

Most participants either agreed or strongly agreed (90.9%) that the education session was an appropriate length of time, and participants either agreed or strongly agreed that it motivated them to increase their physical activity (77.3%), reduce their sitting time whilst not at work (72.7%), and improve their diet (71.2%). When asked in the questionnaire about the key messages they took away from the education session, 41% (*n =* 40) mentioned dietary knowledge changes, and 31% (*n =* 30) mentioned exercise knowledge changes. Participants were happy with the information they received. Despite spending equal time discussing diet and physical activity (90 minutes each), most only recalled learning about diet in interviews more so than other elements such as physical activity, smoking, alcohol, sleep, and mental health.

*“No, it was probably just about right how things were explained and, the little, like game things that they were showing you. How many calories do you think is in this? You know what I mean, the presentation of the games and that were about right, and about the right amount of time.”* [Intervention Participant – M17]

#### Fitbit

Participants either agreed or strongly agreed that the Fitbit increased their awareness of their physical activity (90.5%), sitting time (73.8%) and sleep pattern (88.1%), and it motivated them to make changes to their physical activity (81.0%), and their sitting time (69.0%), but less so their sleep pattern (40.5%). Three quarters (75.4%) of participants engaged in the step challenges, 59.4% either agreed or strongly agreed they were motivating, and 54.7% of participants either agreed or strongly agreed that participating in the step challenges helped to increase their step count. There was less agreement on the step challenges with some participants liking the competition, whilst others did not like competing with “strangers”. Some participants reported the free Fitbit was the main motivation for taking part.

*“Interviewer: So why did you guys decide to take part in the SHIFT study?*

*Participant: Truthful answer … the free Fitbit”* [Intervention Participant – P01, FG1].

The Fitbit was also mentioned as a useful tool for goal setting and a consistent sense of achievement.

*“I do my best to hit all the targets and more on that, I get focused on that. If I haven’t hit all the greens on it if you like, then I will go out of my way to do it, I’ll go for a walk, I’ll do it … because of that I will do it.”* [Intervention Participant – P01, FG1].

#### Text messages

Participants either agreed or strongly agreed that the frequency (84.5%) and content (91.1%) of the text messages was appropriate and informative. The interviews showed that the text messages were good at providing logistical information for the upcoming tasks, and it was beneficial to know that the SHIFT team were readily contactable.

*“They were sent as they were needed, you know it wasn’t an overload for the brain or anything … Any time I actually messaged you I did get a reply back … I knew I was doing something at my end, and you’s were taking it serious at your end … You were always there, because you said if you need us, we’re there. You kept your word.”* [Intervention Participant – M24].

Though if the messages were to be used in a motivational capacity, more frequent and personalised messages would have been beneficial.

*“The only thing the only thing I could think of personally, would be to just ramp them up a little bit more. You know what I mean … Because they were quite well spaced out. You know what I mean. If I’m right, there were only about two of them weren’t there?”* [Intervention Participant – M15].

#### Health assessments (baseline, 6 and 16-18 months)

Though the health assessment was not part of the intervention, most intervention participants either agreed or strongly agreed (87.8%) that the first health assessment motivated them to want to change aspects of their lifestyle, including increasing physical activity (73.2%) or improving diet (70.7%). Fewer control participants either agreed or strongly agreed (63.6%) that the first health assessment motivated them to want to change aspects of their lifestyle, including increasing physical activity (49.5%) or improving diet (52.5%).

Drivers mentioned it was eye-opening to see the results.

*“But when you see it wrote down in front of you, it sort of clicked something in the back of your mind, thinking ‘oh God’... Numbers look a lot worse than looking at yourself because you’re used to looking at yourself …. you look in the mirror and you just tap your belly, and you say ‘blimey I’ve got to lose some weight’. But, when you’re sitting there, and someone writes down, like … I can’t think what it was, it was bleeding high, I know that!”* [Intervention Driver – M15].

Control participants also mentioned it was motivational to improve their health.

*“I didn’t realise I was overweight or, I’ve got a bit of belly but it’s not too big but when they turned around and said you’re obese … and then you’ve got the graph and they point to you in a certain section … Mine wasn’t too bad it was just a little bit above where I should have been so it gave me confidence that I could get there”* [Control Driver – M23].

### Attrition rate within the RCT

Three hundred and eighty-two participants undertook the baseline health assessment, this consisted of 199 control participants and 183 intervention participants. One hundred and forty-five participants (79.2%) took part in the education session. Two hundred and sixty-two participants (68.6%) took part in the six-month follow-up health assessments. The retention rate at 6-months was higher in the control participants (73.9%) than the intervention participants (62.8%), which may be due to intervention participants being ineligible to continue with the study if they failed to attend the education session. Table 4 shows reasons behind discontinuation of the study. A flow diagram of retention rates throughout the study can be seen in the main outcomes paper [12]. Dropout rate in the intervention group may be due to expectations of health improvements not being met.

*“I think that for them that dropped out they just … I think they’d realised that they’d not really made that much of a change, or they made them, but they were short lived if you know what I mean?”* [Intervention Manager – M11].

**Table 4:** Disposition of participants and reasons for discontinuation

		Control, n (%) ***N =*** 199	SHIFT, n (%) ***N =*** 183	Overall, n (%) ***N =*** 382
At baseline	Consented at baseline	199	183	382
	Entered trial and provided data	199	183	382
At 6-months follow-up	Attended and gave data	147 (73.9%)	115 (62.8%)	262 (68.6%)
At 16-18 months follow-up	Completed trial	104 (52.3%)	101 (55.2%)	205 (53.7%)
Discontinued				
Reasons for discontinuation	Participant deceased	1 (0.5%)	0 (0.0%)	1 (0.3%)
	Lost to follow up	61 (30.7%)	51 (27.9%)	112 (29.3%)
	Investigator decision	2 (1.0%)	4 (2.2%)	6 (1.6%)
	Left job	14 (7.0%)	8 (4.4%)	22 (5.8%)
	Long term sickness	5 (2.5%)	2 (1.1%)	7 (1.8%)
	No longer interested	3 (1.5%)	0 (0.0%)	3 (0.8%)
	Cluster withdrawal	13 (6.5%)	15 (8.2%)	28 (7.3%)
	Significant protocol violation	0 (0.0%)	1 (0.6%)	1 (0.3%)
	Suspended from work	0 (0.0%)	1 (0.6%)	1 (0.3%)

Retention rate between the 6-month and final follow up was 78.2%. In this instance, retention was higher in the intervention group (87.8%) than the control group (70.7%). This could be because those who were disinterested in the programme had already dropped out. Those in the intervention group were possibly more invested in the programme, and yet little burden was required of them during this time. In total, from baseline to 16-18 months post-randomisation, 46.3% (*n =* 177) of participants dropped out of the RCT. One of the primary reasons for dropout mentioned by managers was that truck drivers are notoriously transient workers, with a high staff turnover rate. It was evident that a lot of drivers had left their company before the cessation of the programme.

*“Like I say, the drivers that had to drop out, they left, obviously that’s one of those things, they left and went on to different contracts.”* [Control Manager – M12].

Other reasons included long term illness, and a few drivers were isolating, or on furlough.

*“Yeah, a couple on long term sick, and I think a couple that were shielding as well.”* [Intervention Manager – M11].

Another commonly mentioned reason was that some participants saw the timings of the health assessments as inconvenient.

*“They just want to come in and go home. Yeah, they don’t want to do anything else that adds on to their day. Which I totally get.”* [Control Manager – P05].

Finally, two depots (clusters) also withdrew from the trial due to the collapse of their contracting companies.

### Barriers to behaviour change

In the 6-month questionnaires, all participants were open-endedly asked to fill out their main barriers to a healthy lifestyle. Of the 145 responses, 46% suggested their biggest barriers were work-related, predominantly the length, irregularity and start times of their shifts. The second biggest barrier was family-related (12%), such as childcare commitments. This was followed by diet (11%) and self-motivation (10%), with drivers referring to themselves as being “lazy” or “need more discipline”. Table 5 shows a summary of the mentioned health barriers.

**Table 5:** Most frequently mentioned barriers to a healthy lifestyle

Barriers to a Healthy Lifestyle	N (%)
Work-Related	67 (46.2%)
- Long Hours	23 (15.9%)
- Shift Pattern	16 (11.0%)
- Diet at Work	10 (6.9%)
- Lack of Routine	5 (3.4%)
Family	18 (12.4%)
Diet	16 (11.0%)
Self-Motivation	14 (9.7%)
Time (not explicitly work-related)	10 (6.9%)
Weather	4 (2.8%)
Injury/Illness	3 (2.1%)
Sleep	3 (2.1%)
Embarrassed to Exercise in Public	1 (0.7%)

Using an inductive thematic analysis approach in the interview data, we discovered participants discussed barriers to achieving a healthy lifestyle both at work and at home. We deemed it important to mention these barriers as this may influence guidance for future health interventions in this demographic.

Almost all participants mentioned the long, irregular shift patterns and early start times as being a main barrier of a healthy lifestyle.

*“The start times as well, I start between midnight and 4am in the morning and that’s no good for your body rhythm if you like. It’s not good for your body. By 2 o’clock in the afternoon you want to go to sleep.”* [Control Driver – M09].

Lack of managerial support and unrealistic expectations also impacted drivers.

*“It’s not doing one 12-hour shift or one 15-hour shift, it’s one after another, after another, after another. You know, they think you’re machines like these trucks, you can drive them for 24 hours a day, 7 days a week, they’re just trucks, put some more diesel in, put some more oil in, they’ll go forever, we can’t do that. They think we can.”* [Intervention Driver – M14].

Another key barrier included unavailability of healthy foods whilst at work.

*“Well, umm, basically being lorry driver is … the diet is shocking, you can’t really get healthy food it’s all fast food … If I’m parked up somewhere, the only thing that’s available is McDonald’s, or Burger King or sandwiches from WHSmith.”* [Intervention Driver – M14].

Less mentioned, and more debated was the topic of physical activity, with some drivers suggesting they do a lot of physical activity at work, whilst others saying they do not.

*“As lorry drivers, you can’t obviously do thousands upon thousands (of steps) because you might be in your lorry all day. You know what I mean?”* [Intervention Driver – M15].

*“The job side of things … we’re not deficient on exercise, we actually empty our trailers, get out the cab, we empty the backs, we’re physically hands on all the time … at the end of the day we should be built like body builders I think!”* [Intervention Driver – P01, FG1].

Self-motivation was also a key barrier that was mentioned, and this appeared to be relevant both at home and at work.

*“Just being lazy … That’s it. Me just telling myself ‘oi get your big fat ass out of there and go for a walk!’”* [Intervention Driver – M24].

### Perceived behavioural changes

Based on interviews with drivers, it appeared the biggest reported modifications occurred to dietary behaviours, as these were referred to more frequently than physical activity changes.

“*Participant: Well, as I say I cut down my sugars and the saturated fats? I try and do a little bit more exercise although that’s a little bit more difficult haha!*

Interviewer: You mentioned there that exercise was a bit harder to control than the diet, what makes you say that?

*Participant: Well, I mean it’s easy to change from butter to margarine you just buy a different one in the shop, whereas to get your mind to want to go out and do a bit of exercise is a bit harder haha”* [Control Driver – M09].

Significant differences in steps, sitting and standing time only remained significant during non-workdays rather than workdays, this was also reflected in the drivers’ and managers’ opinions about the inherent characteristics of the job.

“*Interviewer: Were the shifts a barrier to actually live the healthy lifestyle, did you just do it around it?*

*Participant 1: Just had to do it around it.*

*Participant 2: It’s more just, not doing it at work, just everything at home”* [Intervention Driver – P01, FG1].

### Suggested improvements to SHIFT

The most frequently suggested improvement from both drivers and managers was that the SHIFT team could have had more regular contact and engagement with the participants throughout the intervention. Participants particularly mentioned that communication dropped off towards the end of the study, when sustainability of the behavioural changes were being tested. However, this was an intentional pause in communication from the SHIFT team’s perspective as part of the extended follow-up period.

*“They were given all the information and then there was quite a long gap, so that was the only negative thing I would say, that gap was too long, they were saying what’s happening and they were starting to lose interest.”* [Intervention Manager – P01].

From the managers’ perspective, more clarity at the beginning would have been helpful in organising and managing expectations.

*“It was a little bit ‘it won’t affect your business, it won’t affect your everyday’ bla bla bla. But it does affect it when it’s 2 or 3 hours and you’ve got to plan that around the drivers’ times, rest days, and all that kind of stuff. That alone, the admin side from myself or one of my managers, to prep it … it’s a few hours in a day, each time, to try and prep that … I don’t mind doing it, but it’s about understanding that’s what is involved.”* [Intervention Manager – M17].

### Future sustainability

All managers were asked if they could see SHIFT becoming sustainable in the future, and if they would support it. All managers said they would support it.

*“Absolutely. 100%. I can say that without even speaking to my, my higher tiers … But I know they’ll be on board, they will, yes … Again, it’s all about the wellbeing of drivers, isn’t it*?” [Control Manager – M12].

Although some managers caveated this with comments about issues with practicalities, especially in smaller sites.

*“Not all drivers would want to participate. Not everybody would want to be part of it. Like I said, in the planning of the drivers and getting the drivers back, it’s sometimes not that easy to be able to manage small contracts and get drivers back on site, the time that you need them.”* [Control Manager – M22].

Truck drivers in the UK must undergo 35 hours of mandatory training every 5 years to maintain their license, called a Certificate of Professional Competence (CPC). Intervention drivers were asked about their thoughts on turning the education session into a CPC module. All drivers responded favourably to this suggestion.

*“I think to have it as part of a CPC I think certainly, would be, be a much easier route and you’d obtain a lot more numbers to be involved.”* [Intervention Manager, M17].

However, almost all drivers said it would need a medical professional rather than a driver trainer to lead the workshop.

*“I think a health professional could present it better than a driver trainer because driver trainers understand driving, but they don’t understand other things …. it’s always better to have an expert talking about something they know, than somebody talking about something they’ve been told to talk about.”* [Intervention Driver – M17].

All drivers said that moving the education session to an app-based workshop would be detrimental but did say that virtual CPC modules have been successful.

*“I mean if you’re in the classroom, with other people and that, then you get involved more, I think if you’re on your own, doing an app, I think a lot of people probably won’t bother.”* [Intervention Driver – M19].

### Control site participants

Control site participants underwent health assessments at baseline and 6-months follow-up. Although these health assessments were only for evaluation purposes, 67.5% of control participants agreed feedback from the health assessments motivated them to change aspects of their lifestyle. There was a lack of awareness from the majority of control site participants and managers as to whether they were in the control or intervention arm, suggesting the SHIFT team could have provided more communication and explanation to both the managers and participants of their allocated arms and what this meant for participants.

*“Interviewer: So were you aware that there were two different groups in the SHIFT study, one was called control and one was called intervention? [Pause] Did you get told that?*

*Participant: No. No, no, no, no. I didn’t know that.”* [Control Driver – M25].

### Contamination

Randomisation occurred at the site level to ensure there was a minimal risk of contamination between intervention and control participants. All participants and managers were asked in the interviews if they had spoken about SHIFT to anybody from any other depot, which they confirmed that they had not.

### SARS-CoV-2 pandemic

A large, unforeseen confounding variable to the RCT was the SARS-CoV-2 pandemic which caused three national lockdowns in the UK between March 2020 to June 2021. Fortunately, almost all (*n =* 254/262, 96.9%) participants had completed the intervention activities and the 6-month follow-up health assessment before the first national lockdown. However, the final follow-up was greatly impacted by this, and as such, all main trial sites required remote data collection, where participants were asked to complete the questionnaires, self-report their weight, wear the activPAL and complete the work diary by themselves. This method was deemed the most appropriate, pragmatic, and safest form of data collection at this time to assess the key main outcomes. When asked in the interviews, it appeared all sites were impacted by the pandemic in different ways. Those who delivered essential items worked busier and longer hours.

*“Manager: In fact, we went through a stage where we were actually really short of drivers.*

*Interviewer: Oh wow, was that due to sickness?*

*Manager: No it was due to operational demand because people were panic buying so our stores we deliver to, were ordering bigger and things like that … Oh lots of hours and double shifts and things like that, just to keep the business going and to keep stocks up and things like that.”* [Intervention Manager – M18].

Some drivers mentioned that it was business as usual.

*“It was the same run on the same start times, so not many changes if you like.”* [Control Driver – M12].

Some drivers mentioned being on furlough for most of the year. However, there was ambiguity between the furlough status across the sites. Several sites furloughed staff on a rotational basis, others furloughed all staff, others made it optional for drivers to choose furlough, and other sites that provided essential goods did not make furlough an option.

*“I was put onto furlough, I was put on that until August … then we were locked down again in November, so we were put on furlough then … Since April I have probably done less than … 10 days’ work?”* [Control Driver – M21].

Some managers also mentioned the pandemic halted the enthusiasm for the study, with priorities being re-evaluated.

*“It’s just after that the communication and the engagement because of COVID-19, where that stopped, then obviously SHIFT stopped as well … well in the drivers’ minds. So they just said ‘it’s not continuing anymore obviously’. Nobody’s ringing us, and everybody else had obviously their personal interests in their minds during this pandemic as well. So they just lost focus on it.”* [Intervention Manager – M10].

## Discussion

### Overall findings from the process evaluation

Overall, SHIFT was delivered as intended to those who were able to attend the education session, with all intervention participants who attended the education session also receiving a Fitbit and cab workout equipment. However, 20.8% of the intervention group did not attend the education session. Furthermore, some discrepancies were seen with the number of participants per education session being lower than intended in the study protocol and the duration between the baseline health assessment and the education session being longer (due to scheduling challenges) than intended in the study protocol. The majority of participants reported being motivated to increase their physical activity, improve their diet and reduce their sitting time following the education session. There was also a reported increase in knowledge and awareness regarding the importance of physical activity and a balanced diet. The Fitbit was the favoured component of the intervention, which is consistent with the literature as a way to promote an increase in physical activity [20]. The cab workout appeared the least favoured and too cumbersome for the majority. Though most intervention participants reported improvements to both knowledge and behaviour of their dietary intake, dietary outcome measures within the effectiveness trial did not support this. The most common suggested improvement of the study was to increase frequency of communication with participants. Barriers to behaviour change were apparent throughout the intervention, specifically the irregularity and long duration of drivers’ shift patterns. Consequently, reported behaviour changes mainly occurred outside of working hours, which was supported by the effectiveness trial. These barriers require a high level of motivation by this at-risk group to change behaviour, therefore regular and sustained contact would be required to maintain improved health behaviours. Managers and drivers reported enthusiasm and necessity for SHIFT to be included in future CPC training. Future health interventions in this workforce should focus more on sleep and diet, in addition to providing more opportunities for physical activity on workdays, as these lifestyle factors appear to be truck drivers’ priorities.

Using the MRC Process Evaluation Framework, the implementation process, the mechanisms of impact which influenced the findings, and the contextual factors which may have affected the RCT are discussed below.

### Implementation

The education session was regarded as valuable by all interviewed intervention participants, and increased perceived knowledge, particularly about healthy diets. However, only 79.2% (145/183) of intervention participants attended the education session, due to logistical challenges and operational requirements. This shows that although the education session was beneficial, it was not wholly feasible in this hard-to-reach occupational group, with key issues being the varying start times, operational demand, and time-critical deliveries. Having said this, attendance at the education session is in line with similar studies in occupational groups [17, 21]. Additionally, in a real-world setting, only 15-30% of people with newly diagnosed diabetes in the UK attend NHS organised education sessions, despite high referral rates from general practitioners [22]. Therefore, although challenging to organise, embedding health education sessions into the workplace could substantially improve the reach to at-risk occupational groups.

The SARS-CoV-2 pandemic has seen NHS settings offer virtually delivered group-based sessions which may help to increase future reach/uptake. SHIFT education sessions delivered virtually should be explored as part of future implementation research, which could provide more flexibility for drivers and potentially increase uptake and overcome some of the barriers experienced within this trial linked to the scheduling of the education sessions. Within the RCT, organising times for the drivers to attend the education session took longer than anticipated which may have impacted the results at the 6-month follow-up.

The Fitbit was regarded an important tool for increasing understanding of current activity levels, and for providing regular motivation for increasing daily step counts. There was high adherence to the Fitbit, suggesting it was an effective tool for providing participants with a method of monitoring their activity levels. The cab workout equipment was less used by participants, with the biggest reason being that there were more important priorities whilst on breaks (i.e., eating and sleeping). Some participants used the cab workout equipment, and 16.3% agreed it increased overall levels of physical activity. Adherence to the cab workout appeared low, therefore the cab workout has been regarded as a poor tool to encourage behaviour change. Future research into this occupational group should address eating behaviours and sleep, given these behaviours were highlighted as priorities by drivers. Improvements in sleep quality and duration in drivers, may create more opportunities for physical activity.

### Mechanisms of impact

**Fig. 2:**
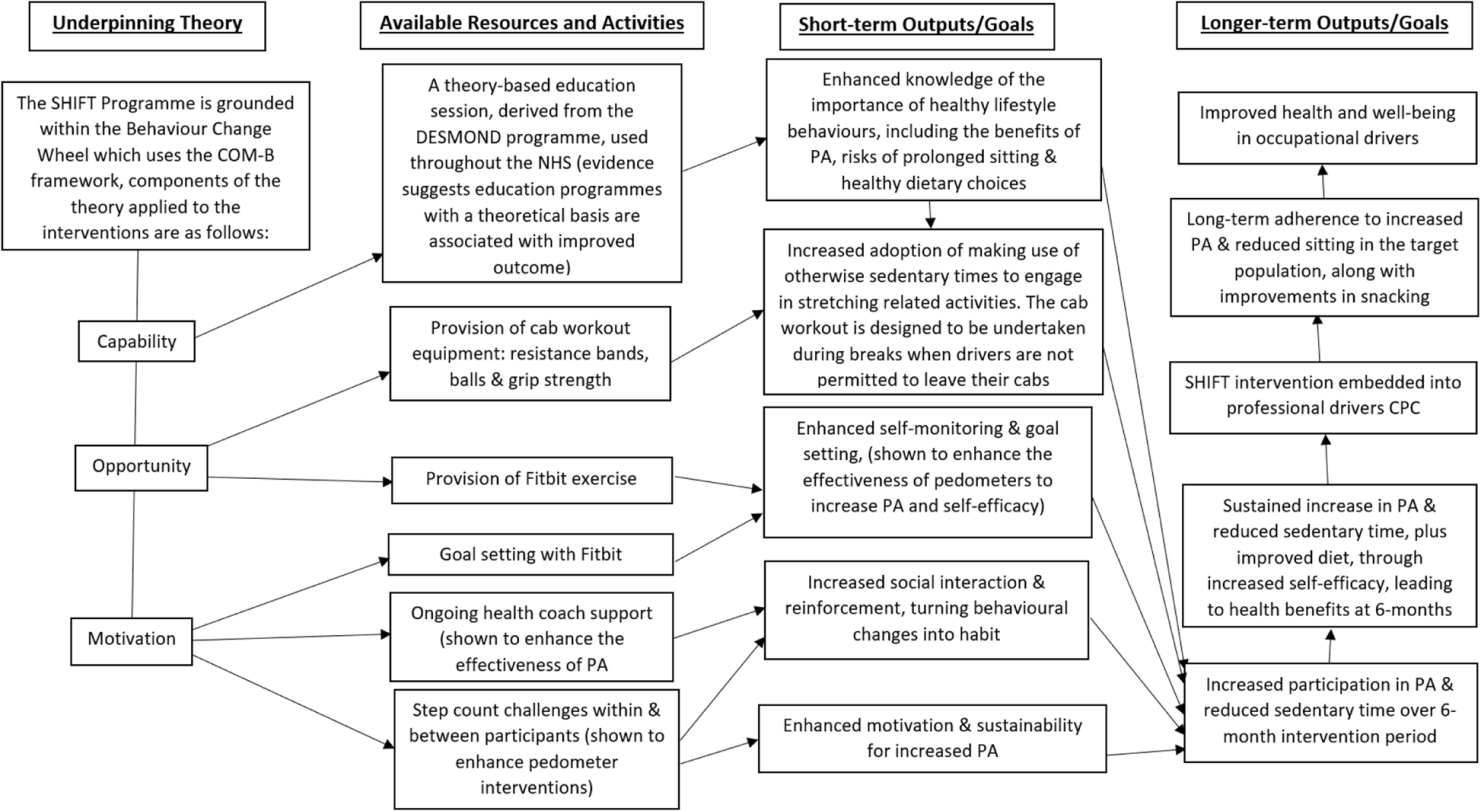
Refined SHIFT logic model following the process evaluation

The intervention used Bandura’s SCT as the theory of behaviour change [14]. SCT suggests that learning can occur through observing and imitating someone else’s behaviour. It is most effective when the observer witnesses a model with similarities (such as another truck driver) carrying out the behaviour. It focuses on the triadic model where personal factors, environmental influences, and behaviour continually interact [14]. Bandura suggests the key concepts which affect health behaviour change interventions include self-control, self-efficacy, observational learning, and reinforcement. Based upon the SHIFT logic model, self-efficacy and self-monitoring elements were to be utilised with the Fitbit [11]. The supportive social environment was facilitated via health coach support with the text messaging service and recruitment of local champions. Acquisition of the essential knowledge relating to behaviours came from the education session. However, in SHIFT, truck drivers are inherently isolated from each other, limiting interactions and reducing opportunities to learn behaviours from each other, which is a key component to SCT. In addition, the education sessions consisted of fewer drivers than originally intended, reducing the opportunity to increase one’s self-efficacy through vicarious learning. An important evaluation outcome, as recommended by the MRC, is a refined programme theory to examine alternative theories to best inform transferability of the intervention across settings [23]. The model which would fit best with the behaviour changes seen from SHIFT would be the Behaviour Change Wheel which uses the COM-B framework, where participants require capability, opportunity, and motivation to change their health behaviours [24]. The updated proposed logic model is seen in Fig. 2.

There was a significant difference in step counts in favour of the intervention group compared to the control group at 6-months follow-up. As this was the primary outcome, it shows the intervention was effective. Dietary changes such as fruit and vegetable intake and dietary quality score were unchanged between baseline and 6-months, despite the fact multiple interviewed participants suggested dietary intake was the biggest change made. Drivers also suggested it was easier to change diet than increase physical activity, which is consistent with other process evaluations of workplace health promotion interventions [25]. The discrepancy between the process evaluation and effectiveness trial findings relating to dietary behaviours could be due to the limitations of the FFQ (used as an outcome measure in the RCT). Previous studies show poor validation of an FFQ compared to a 4-day weighed food record [26]. It also is possible that participants in the intervention group may have altered their intentions towards dietary intake, but this may have not translated into habitual behaviour change. Changes in policy, such as improved access to healthier foods at truck stops, may be required to create behavioural changes related to diet in truck drivers. Future research should look to implement changes in policies at the organisational and environmental levels, which in turn may facilitate improvements in dietary intake in this population.

### Context

All participants were asked in the 6-month questionnaire about any major changes to their life throughout the duration of the study – these included moving house, family illness and relationship breakup. There were no apparent biases between groups regarding external factors influencing participation.

The SARS-CoV-2 pandemic had a major impact on the study overall. Although there appeared to be no systematic differences between intervention and control sites when interviewed, it was a rapidly changing, dynamic situation that was unable to be adequately reported. Participants completed a furlough questionnaire (*n =* 146) at 16-18 months follow-up which revealed that more control participants had been furloughed than intervention participants (58.1% vs. 23.7%, respectively). We can therefore not say with certainty that there were no differences in the impact of the SARS-CoV-2 pandemic between groups.

Study outcomes were measured using health assessments, which all intervention and control participants attended. These were followed by short feedback sessions where the results were explained to each participant. Although not part of the intervention, these health assessments did reportedly have an impact on awareness and knowledge about a healthy lifestyle in both groups, which is consistent with other research [17]. This was an unintended outcome of the study, which although did not lead to behavioural changes in control participants, did provide participants with a more holistic understanding of their current health status.

Organisational readiness is key for both research and practice in this area. Future recommendations include detailed meetings with all levels of management such as site managers, human resource staff, driver trainers, and regional managers to understand what resources (including time) will be expected from them and at what stages.

### Study strengths and limitations

The triangulation of data led to a more comprehensive understanding and rigorous analysis, as we were able to capture data using different dimensions of the same phenomenon [27]. Data were collected at multiple levels, including the drivers, their managers, and site-level data to provide a more complete understanding specifically of the context incorporating the RCT. Process evaluation data were collected from baseline to the completion of the study (16-18 months follow-up), this allowed us to follow the participants’ reflections throughout. The length of follow up at the end gives the participant and managers time to reflect and provide more holistic responses of their experiences. The representativeness of each depot was considered when stratified sampling of drivers and managers for the interviews took place. This method gives the reader a more thorough comprehension of the study, as sites were heterogenous. The process evaluation was undertaken primarily by a single integrated evaluator, which was beneficial for effective communication, avoided duplication of effort and reduced participant burden [15]. Very much part of the intervention team, the evaluator used this first-hand experience to thoroughly understand every part of the intervention. This in turn helped to minimise the Hawthorne effect [28], whilst collecting observational data about the operational challenges for both for the implementation team and the depots. Providing a balanced process evaluation of the SHIFT RCT offers important lessons that may inform the future development of health interventions for drivers [29].

Assessing the reach of SHIFT across all depots was not appropriate or feasible within the context of the programme, as the study aimed to recruit approximately 14 participants per site due to funding constraints. It was apparent that in most sites, there was a higher interest than the maximum recruitment level, and as such, highlights the necessity of these health interventions in this at-risk population. The total loss to final follow-up from the study was high (46.3%), resulting in potential attrition bias where there may have been systematic differences between completers and non-completers. Multiple baseline characteristics were checked such as age, BMI, total daily steps, which resulted in no observed differences, although non-completers were more likely to be from smaller sites, which may suggest extra barriers within smaller sites. All main trial participants were invited to participate in interviews and incentivised to do so, which may have led to sampling bias. The limitation of having an integrated process evaluator is increased risk of researcher bias, which was mitigated through having an external critical friend (AC) and all findings discussed with the principle investigator (SC) [30]. As the process evaluation data were analysed without the knowledge of the trial outcome, the bias was also minimized so as to reduce influenced interpretations.

## Conclusions

The RCT of SHIFT showed that the intervention group had higher step counts at 6-months follow-up than the control group due to the control group reducing their step counts and the intervention group remaining stable. Although the participants valued the intervention (particularly the education and the Fitbit), the participants mostly reported relaying dietary focused key messages from the education session and viewed dietary changes more highly than physical activity changes. This may explain why no increases in physical activity were found. Future extension of this study should consider better assessment of nutritional intake to capture potential dietary behavioural changes, as regularly reported in the interviews and questionnaires. One of the biggest barriers for the cab workout was that drivers prioritised sleeping on their breaks. Future work should look at improving sleep duration and quality in drivers, which in turn will provide more opportunities for exercising during breaks. More frequent contact with both control and intervention participants was suggested as the key improvement, which would lessen attrition. Attrition rates were high throughout the study, which supports previous understanding that truck drivers are a hard-to-reach population, not least due to the transient nature of the job. SARS-CoV-2 had a mixed impact on participating sites, which would make any conclusions about the final follow-up uncertain. Overall, participants were enthusiastic about SHIFT, with particular emphasis on the dietary knowledge gained from the education session and the activity monitoring and motivation from the Fitbit. The integration of SHIFT into a CPC module was regarded with widespread support from both managers and drivers, this would allow scalability of the intervention by providing access to all UK truck drivers.

## Data Availability

The datasets used and/or analysed during the current study are available from the corresponding author on reasonable request.

## Abbreviations

CPC: Certificate of Professional Competence
FFQ: Food Frequency Questionnaire
MRC: Medical Research Council
NIHR: National Institute of Health Research
RCT: Randomised Controlled Trial
SCT: Social Cognitive Theory
SHIFT: Structured Health Intervention For Truckers
UK: United Kingdom
